# Assessing the Accuracy and Reliability of Large Language Models in Psychiatry Using Standardized Multiple-Choice Questions: Cross-Sectional Study

**DOI:** 10.2196/69910

**Published:** 2025-05-20

**Authors:** Kaitlin Hanss, Karthik V Sarma, Anne L Glowinski, Andrew Krystal, Ramotse Saunders, Andrew Halls, Sasha Gorrell, Erin Reilly

**Affiliations:** 1 Department of Psychiatry and Behavioral Sciences University of California, San Francisco San Francisco, CA United States

**Keywords:** artificial intelligence, mental health, digital mental health, knowledge assessment, AI

## Abstract

**Background:**

Large language models (LLMs), such as OpenAI’s GPT-3.5, GPT-4, and GPT-4o, have garnered early and significant enthusiasm for their potential applications within mental health, ranging from documentation support to chat-bot therapy. Understanding the accuracy and reliability of the psychiatric “knowledge” stored within the parameters of these models and developing measures of confidence in their responses (ie, the likelihood that an LLM response is accurate) are crucial for the safe and effective integration of these tools into mental health settings.

**Objective:**

This study aimed to assess the accuracy, reliability, and predictors of accuracy of GPT-3.5 (175 billion parameters), GPT-4 (approximately 1.8 trillion parameters), and GPT-4o (an optimized version of GPT-4 with unknown parameters) with standardized psychiatry multiple-choice questions (MCQs).

**Methods:**

A cross-sectional study was conducted where 3 commonly available, commercial LLMs (GPT-3.5, GPT-4, and GPT-4o) were tested for their ability to provide answers to single-answer MCQs (N=150) extracted from the Psychiatry Test Preparation and Review Manual. Each model generated answers to every MCQ 10 times. We evaluated the accuracy and reliability of the answers and sought predictors of answer accuracy. Our primary outcome was the proportion of questions answered correctly by each LLM (accuracy). Secondary measures were (1) response consistency to MCQs across 10 trials (reliability), (2) the correlation between MCQ answer accuracy and response consistency, and (3) the correlation between MCQ answer accuracy and model self-reported confidence.

**Results:**

On the first attempt, GPT-3.5 answered 58.0% (87/150) of MCQs correctly, while GPT-4 and GPT-4o answered 84.0% (126/150) and 87.3% (131/150) correctly, respectively. GPT-4 and GPT-4o showed no difference in performance (*P*=.51), but they significantly outperformed GPT-3.5 (*P*<.001). GPT-3.5 exhibited less response consistency on average compared to the other models (*P*<.001). MCQ response consistency was positively correlated with MCQ accuracy across all models (*r*=0.340, 0.682, and 0.590 for GPT-3.5, GPT-4, and GPT-4o, respectively; all *P*<.001), whereas model self-reported confidence showed no correlation with accuracy in the models, except for GPT-3.5, where self-reported confidence was weakly inversely correlated with accuracy (*P*<.001).

**Conclusions:**

To our knowledge, this is the first comprehensive evaluation of the general psychiatric knowledge encoded in commercially available LLMs and the first study to assess their reliability and identify predictors of response accuracy within medical domains. The findings suggest that GPT-4 and GPT-4o encode accurate and reliable general psychiatric knowledge and that methods, such as repeated prompting, may provide a measure of LLM response confidence. This work supports the potential of LLMs in mental health settings and motivates further research to assess their performance in more open-ended clinical contexts.

## Introduction

Over the past decade, there has been a significant surge of interest in the application of artificial intelligence (AI), particularly large language models (LLMs), within medical contexts. While AI-related efforts in medicine have historically focused largely on applying deep learning methods to analyze data, most notably image data [1,2], more recent advancements at the intersection of natural language processing, deep learning, and generative AI have produced LLMs capable of generating and interpreting complex clinical text [3-5]. These developments have prompted inquiry among developers and researchers about the ability of LLMs to assist in a range of pressing medical tasks, including increasing the efficiency of clinical documentation, supporting clinical decision-making, and developing educational patient simulations [6]. In psychiatry and mental health, the text generation and interpretation capabilities of LLMs have generated enthusiasm for their potential applications in screening and diagnosing psychiatric illnesses, generating risk assessments, and serving as therapeutic chatbots [7-9]. Amid the severe shortage of psychiatric providers [10,11], these envisioned LLM tools could increase provider efficiency and offer new modalities for treatment, addressing critical gaps in mental health care equity and access.

However, despite their potential benefits, employment of LLMs in the broader domain of mental health carries significant potential risks, such as producing inaccurate, unreliable, or biased responses, raising concerns about their safety and efficacy, especially in a field already besieged by stigma. Under the hood, LLMs, such as ChatGPT models from OpenAI, Large Language Model Meta AI (LLaMA) from Meta, and Claude from Anthropic, are deep neural networks (typically transformer architectures) with billions to trillions of parameters that have been trained on a massive corpus of unstructured text, including webpages, books, and video transcripts [3,4,12-14]. The “knowledge” these models produce can be divided into two distinct forms: (1) parametric knowledge, which consists of the information encoded in the model’s weights during pretraining and (2) explicit knowledge, which is presented to the model after the training process (eg, through the user’s prompt or a retrieval-augmented system). While explicit knowledge can be updated or changed rapidly, parametric knowledge is encoded in the model and changes only when the entire model is retrained. As is true of all predictive models, LLMs may encode bias reflected in their training data and are limited to the knowledge contained in training examples, which can manifest in inaccurate or unreliable performance and can contribute to their potential for harm. For example, a recent study found that GPT-4’s clinical scenario responses are influenced by societal biases, causing it to recommend erroneous diagnoses and management plans based on factors such as race and gender [15]. Other studies have consistently shown that LLMs may misinterpret specialized terminology (eg, “egosyntonic”) within domain-specific text [16,17].

Given these demonstrated potential risks, the successful deployment of LLMs for mental health tasks will require close attention to (1) the quality of mental health information in their underlying training data, (2) the resulting accuracy of the psychiatric parametric knowledge, that is, the “knowledge” stored within the models’ parameters after training, and (3) the reliability with which the models produce accurate psychiatric answers. In addition, to promote the responsible use of LLM-based systems, it will be essential to develop methods to quantify the level of confidence that can be placed in LLMs’ responses.

To evaluate the medical parametric knowledge encoded in LLMs, researchers in various subfields of medicine have assessed the accuracy of LLM answers to standardized multiple-choice questions (MCQs) from examinations commonly used for medical licensing or education [18-20]. Generally, investigations of LLM performance on a range of examinations, such as the United States Medical Licensing Exam, have reported accuracy rates surpassing those of qualified human test takers [18-20]. However, findings of investigations across different subfields of medicine are variable [18], highlighting a need to characterize performance in different domains and clinical contexts. Notably, no work to date has characterized LLM performance in psychiatry knowledge assessments [18]. Characterizing LLM performance in psychiatric contexts is especially important, as these models may be particularly vulnerable to inaccuracies or biases in mental health clinical contexts. Specifically, there is a significant volume of mental health–related misinformation on the internet [21,22], and there are well-documented challenges with the reliability and validity of psychiatric diagnoses [23,24]. Thus, LLMs may encode inaccurate information drawn from unreliable online sources or reflect underlying clinical uncertainties, making it critical to rigorously evaluate their performance.

In addition to accuracy, there is a need to investigate LLM’s reliability in answering psychiatric questions and develop measures of “confidence” in their responses. An essential property of any autonomously operating LLM tool, such as a patient-facing mental health chatbot, is the ability to *reliably* provide accurate and appropriate responses. Furthermore, incorporating a measure of response confidence may be crucial for ensuring safety guarantees or helping providers and patients contextualize and interpret LLM outputs.

To date, most research exploring LLM performance on standardized MCQs has focused on the popular and commercially available GPT family of models, colloquially known as “ChatGPT” [18-20]. While this family of LLMs is rapidly expanding, common models include GPT 3.5, 4, and 4o. GPT-3.5, released in November 2022, contains approximately 175 billion parameters and shows a significant improvement over its predecessor (GPT-3) by using reinforcement learning from human feedback training to enhance its ability to follow instructions and maintain coherent conversations [25,26]. GPT-4 was subsequently released in March 2023. It introduced multimodal capabilities, enabling it to process both text and images, and displayed better performance in complex tasks and standardized tests (eg, Scholastic Assessment Test, Graduate Record Examination, and bar exams) [27]. Although its exact parameter count has not been publicly disclosed, it is widely speculated to have approximately 1.8 trillion parameters [4,26]. GPT-4o (or GPT-4 “omni”), introduced shortly thereafter in November 2023, further expanded multimodal capabilities to speech and claimed to achieve the same performance as GPT-4 but at 50% reduced cost and greater speed [28]. While its underlying architecture has not been publicly disclosed, there is speculation that it may contain fewer parameters than GPT-4 or may have been trained on a smaller, more curated dataset [26].

To our knowledge, no comprehensive evaluation of the psychiatric parametric knowledge encoded in commercially available LLMs has been published to date. Similarly, there is a gap in the literature regarding the assessment of LLM reliability and the identification of predictors of LLM response accuracy within medical domains. To address these gaps, this study focuses on evaluating the accuracy and reliability of LLMs in psychiatry and attempts to identify the predictors of answer accuracy through the following three aims:

1. To evaluate the psychiatric knowledge encoded in 3 commonly available LLMs (GPT-3.5, GPT-4, and GPT-4o) by assessing their performance on standardized psychiatry MCQs. Although performance depends on many factors, past work has suggested that models with more parameters achieve superior performance [29]. Therefore, we hypothesized that models with significantly more parameters would perform better (ie, GPT-4 would outperform GPT-3.5). In addition, OpenAI claims that GPT-4o maintains the performance of GPT-4 on routine tasks but may be less optimal for specific edge cases [27,28]. Because answering psychiatry MCQs seems more niche compared to other tasks required by GPT models, we further hypothesized that GPT-4o, due to its optimization, may perform slightly worse than GPT-4 but still better than GPT-3.5.
2. To analyze the reliability of these LLMs in psychiatric assessments by examining response variance for the same MCQ over 10 trials. We hypothesized that models with greater accuracy would exhibit more consistency in their responses (ie, higher variance). Based on our hypotheses on accuracy above, we hypothesized that the order of consistency is as follows: GPT-4 > GPT-4o > GPT-3.5.
3. To explore two predictors of LLM accuracy in response to MCQs: (1) the model’s self-reported confidence for the MCQ and (2) the model’s response consistency for the MCQ. We hypothesized that there would be no significant correlation between the model’s self-reported confidence and the accuracy of the response. We anticipated that the model would be more likely to generate accurate responses to a MCQ when it demonstrated greater response consistency across multiple attempts for that same question.

This work represents essential foundational research for the integration of AI into mental health care, evaluating how models like GPT-3.5, GPT-4, and GPT-4o encode and apply psychiatric knowledge. Through a systematic exploration of LLM performance for standardized psychiatry MCQs, we highlight the current capabilities of these models and outline considerations for their safe and effective use in clinical settings.

## Methods

### Models

A total of 3 LLMs (GPT-3.5, GPT-4, and GPT-4o) were selected for this evaluation. GPT-3.5 is an LLM with 175 billion parameters [26]. GPT-4 is estimated to have 1.8 trillion parameters (unconfirmed), while GPT-4o is a faster, more efficient version of GPT-4 with an unknown parameter count [26]. These models were selected due to their widespread commercial availability, extensive user base, and common use in clinical informatics settings [19]. In addition, our institution maintains a Health Insurance Portability and Accountability Act–compliant AI ecosystem that allows these models to be accessed under a license that ensures adherence to regulatory standards [30]. Importantly, under this license, data used in this study were not stored by OpenAI and may not be used to train future LLMs [30].

For analysis, “model temperature” and “top_p” parameters were set to 0.6 and 0.7, respectively, in line with OpenAI guidance for “exploratory code writing” [31], which we believed, a priori, would offer sufficient determinism and flexibility for the MCQ task at hand.

### Dataset

**Figure 1:**
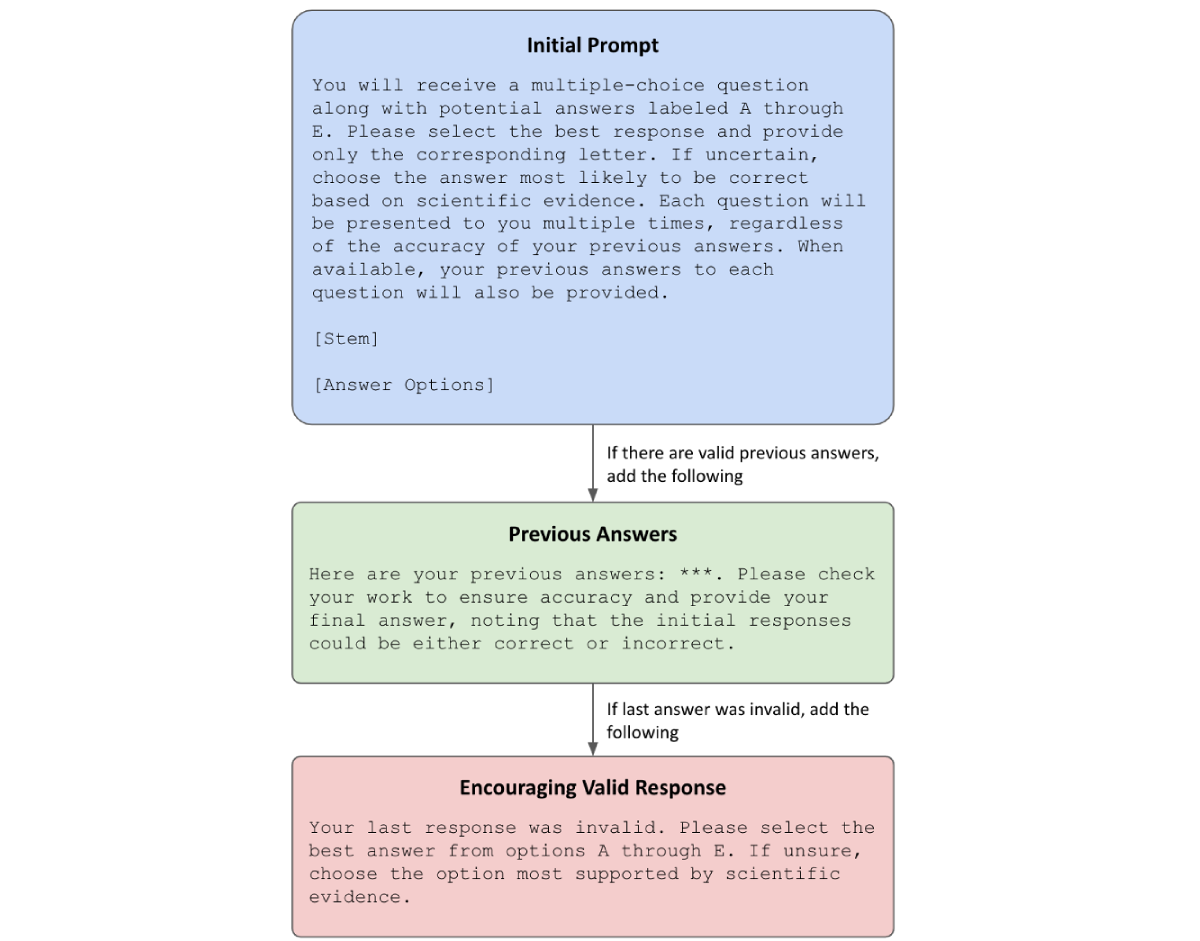
Answer prompting. Zero-shot prompting schema for requesting large language model answers to multiple-choice questions.

A total of 150 single-answer MCQs were extracted from a practice test in the *Psychiatry Test Preparation and Review Manual E-Book*, a comprehensive textbook for psychiatry physicians preparing for the American Board of Psychiatry and Neurology’s certification [32]. Each MCQ included a question stem, 5 answer options (A through E), a correct answer, and a question domain (eg, psychopharmacology and neuroscience). To ensure consistency and reduce confounding, all MCQs were standardized using a uniform format. Questions were encoded to be uniform in structure (ie, stem followed by answer options), using a multiple-choice single-answer format without forced justification [33], and prefaced with a standard prompt explaining the MCQ task (Figure 1).

Of note, the MCQs are designed to cover general psychiatric knowledge but do not comprehensively evaluate subfields of psychiatry such as child and adolescent psychiatry or geriatric psychiatry. In addition, the *Psychiatry Test Preparation and Review Manual E-Book* was published online and publicly available on March 31, 2020, making it possible that its contents were included in the training data for all GPT models. Since OpenAI has not disclosed the training sources, this cannot be confirmed.

### Prompting Procedure

#### Answering MCQs

We evaluated the performance of 3 OpenAI LLMs: GPT-3.5, GPT-4, and GPT-4o. Each model was programmatically instructed to answer every MCQ 10 times to allow for assessment of both accuracy and response consistency. Using a zero-shot prompting approach [34], the initial prompt for each MCQ presented the question stem and answer options and instructed the model to answer the question based on the best available scientific evidence. We opted to not use more advanced prompting optimization techniques, such as role prompting, few-shot learning, and chain-of-thought reasoning, because we believed these to be less face valid for capturing the behavior of a typical user (ie, patient or provider) in interacting with LLMs. Future work may explore how to optimize prompts to yield the best possible outputs.

For subsequent prompts, the model was also provided with a list of its previous answers. Importantly, this design introduced intentional variability across queries to facilitate inconsistency within a limited number of trials. Employing a larger number of trials was neither feasible for this study nor cost-effective or practical for future applications that rely on response consistency as an estimate of confidence in model-generated answers.

Building on methods described in the study by Wang et al [35], all models were given a maximum of 15 attempts to provide 10 valid responses to every MCQ (defined as answering with a letter option A through E). Responses were assessed programmatically for their validity, and following the model responding with an invalid response (eg, “H”), the prompt was amended to offer additional encouragement to adhere to valid responses (ie, to answer with a letter “A” through “E”).

Answer prompting is presented in Figure 1. Multimedia Appendix 1 provides example prompts and GPT responses.

#### Generating LLM Self-Reported Confidence

When prompted directly, many LLMs will assign numerical confidence values to their responses. To evaluate this phenomenon in psychiatry MCQs, we adapted prompting schemes established by Xiong et al [36] to ask the models to report self-confidence. Models were presented with the MCQ stem and answer options and instructed to rate the likelihood from 1 to 100 that it would be able to produce a response that was both accurate and relevant. Preanswer evaluation (as opposed to asking the model to rate its confidence in its answer or other approaches) was chosen to evaluate the models’ a priori self-reported confidence. All models were given a maximum of three attempts to provide 1 valid response defined as a response that contained a number ranging from 1 to 100. Following an invalid response, the prompt was amended to encourage valid output.

**Figure 2:**
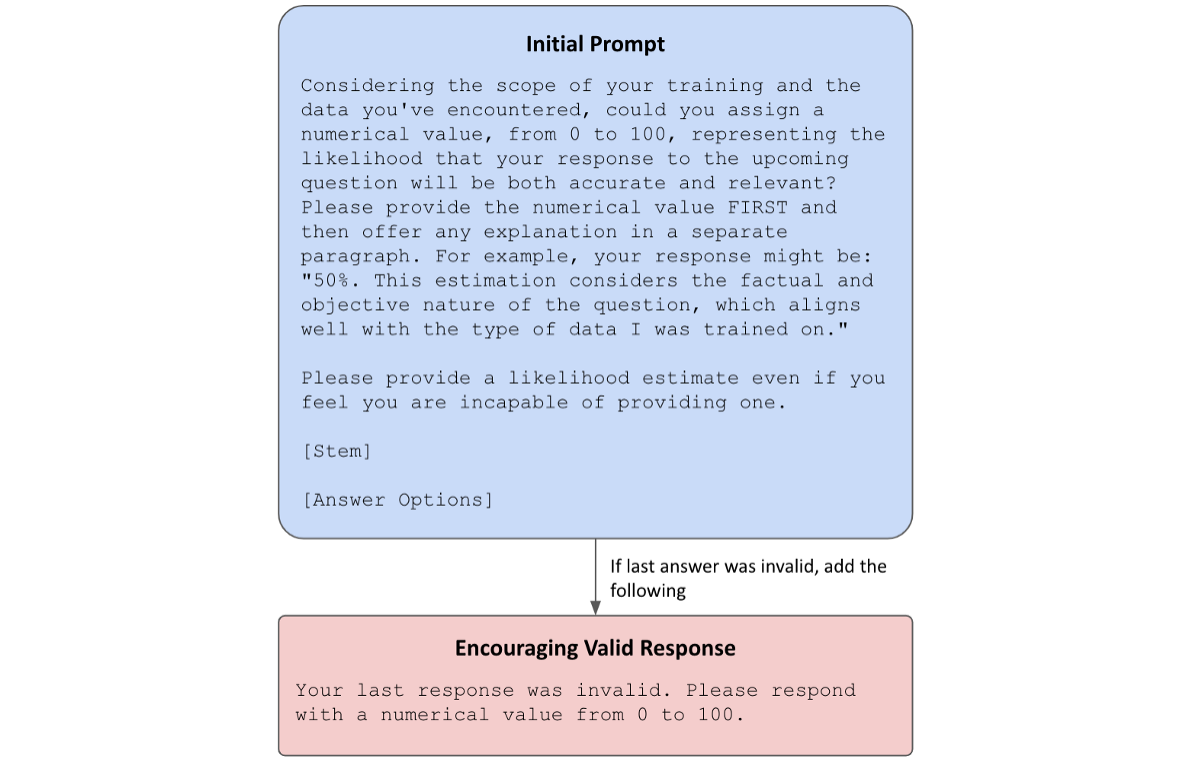
Self-confidence prompting. Zero-shot prompting schema for requesting large language model self-confidence for answers to multiple-choice questions.

Self-confidence prompting is presented in Figure 2. Multimedia Appendix 1 provides example prompts and GPT responses.

### Statistical Analysis

#### Model Accuracy

To simulate real-world scenarios (ie, in which providers and patients would most likely only ask an LLM a question once, for instance, “What medication for depression is associated with the least QT prolongation?”), accuracy was defined as the proportion of MCQs a model answered correctly on its very first (ie, first of 10) attempt. Chi-square tests were used to compare accuracy between models with α=.01 after Bonferroni correction for multiple hypothesis testing.

#### Response Consistency (Reliability)

To evaluate response consistency across trials, we first calculated the distribution of answers for each MCQ and model. Specifically, for every MCQ and model, we recorded what proportion of the time each answer (A through E) was chosen over the 10 question-answering trials, resulting in a frequency distribution of responses. We then calculated response consistency using the variance of these frequency distributions:


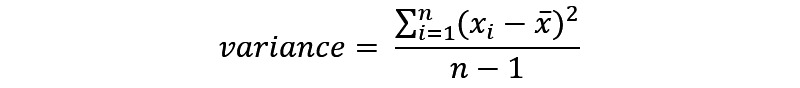


For example, if the model answered “A” consistently across multiple trials, this would yield a frequency distribution of {A: 1, B: 0, C: 0, D: 0, E: 0} and a variance of 0.2. In contrast, if the model answered “A, A, B, B, C, C, D, D, E, E,” this would yield a frequency distribution of {A: 0.2, B: 0.2, C: 0.2, D: 0.2, E: 0.2} and a variance of 0. Thus, response consistency ranges from 0 to 0.2 and can be interpreted as follows: a higher response consistency indicates that the model selected more consistent answers to the MCQ (ie, 1 option was chosen with high frequency and the others with low frequency), and a lower response consistency indicates that the model frequently changed its answer to the MCQ. We used *t* tests to compare average response consistency across MCQs between models with α=.01 after Bonferroni correction.

#### Predictors of Model Correctness

We investigated two potential predictors of model accuracy: (1) response consistency and (2) the model’s self-reported confidence. To examine whether questions with higher response consistency were more likely to be answered correctly by the model, point-biserial correlation coefficients were calculated between the model’s response consistency to each MCQ and the model’s accuracy in answering that MCQ. Similarly, we calculated point-biserial correlation coefficients between the model’s self-reported confidence for each MCQ and its correctness. Statistical significance was determined using an α level of .01, which was adjusted with Bonferroni correction for multiple hypothesis testing.

## Results

### Model Accuracy

On the first attempt, GPT-3.5 answered 58.0% (87/150) of MCQs correctly, whereas GPT-4 and GPT-4o answered 84.0% (126/150) and 87.3% (131/150) correctly, respectively (Table 1). Chi-square tests confirmed that both GPT-4 and GPT-4o outperformed GPT-3.5 significantly (*P*<.001). There was no substantial difference between the performances of GPT-4 and GPT-4o (N=150; χ^2^_1_=0.434; *P*=.51).

**Table 1:** Model accuracy.

Model	Correct answers^a^ (N=150), n (%)	Chi-square test results					
		GPT 3.5		GPT 4		GPT 4o	
		Chi-square (*df*)	*P* value^b^	Chi-square (*df*)	*P* value^b^	Chi-square (*df*)	*P* value^b^
GPT-3.5	87 (58.0)	—^c^	—	—	—	—	—
GPT-4	126 (84.0)	23.4 (1)	<.001	—	—	—	—
GPT-4o	131 (87.3)	31.0 (1)	<.001	0.434 (1)	.51	—	—

^a^Model accuracy is assessed by the percentage of multiple-choice questions answered correctly. Note that accuracy was based solely on the model’s first attempt at the multiple-choice question.

^b^Chi-square *P* values compare accuracy between models with α=.01 after Bonferroni correction.

^c^Not applicable.

### Response Consistency (Reliability)

Across 10 trials for every MCQ, there were significant differences in the response consistencies of the 3 models. Compared to GPT-4 and GPT-4o, GPT-3.5 exhibited significantly less response consistency (Table 2). In addition to being more consistent on average, GPT-4 and GPT-4o appeared to more often offer the same answer across all trials (Multimedia Appendix 2). Among questions that GPT-3.5 answered correctly, it chose the same answer across 10 trials only 10% of the time. Among questions that GPT-3.5 answered incorrectly, it never chose the same answer across all 10 trials. In contrast, GPT-4 and GPT-4o offered the same answer across trials to 90% and 82% of questions answered correctly, and 25% and 26% of questions answered incorrectly, respectively (Multimedia Appendix 2). There were no significant differences between the response consistencies of GPT-4 and GPT-4o (*P*=.36) (Table 2).

**Table 2:** Model consistency.

Model	Consistency, mean (SD)	*t* test results^a^							
		GPT 3.5			GPT 4			GPT 4o	
		*t* test (*df*)	*P* value	*t* test (*df*)		*P* value	*t* test (*df*)		*P* value
GPT-3.5	0.082 (0.0549)	—^b^	—	—		—	—		—
GPT-4	0.182 (0.0394)	18.0 (298)	<.001	—		—	—		—
GPT-4o	0.186 (0.0324)	19.6 (298)	<.001	0.917 (298)		.360	—		—

^a^*t* tests were used to compare average response consistency between models with α=.01 after Bonferroni correction.

^b^Not applicable.

### Predictors of Model Correctness

As suggested by the reliability results above, response consistencies for all models displayed significant, positive correlations with response correctness (Table 3; *P*<.001). Point-biserial correlation coefficients indicated that GPT-3.5’s response consistency was moderately correlated with correctness (*r*=0.304; *P*<.001). The response consistencies of GPT-4 and GPT-4o were strongly correlated with correctness (*r*=0.682; *P*<.001 and *r*=0.590; *P*<.001, respectively).

**Table 3:** Response consistency as a predictor of correctness.

Model	Consistency, mean (SD)		Correlation coefficient^a^	*P* value^b^
	Correct responses	Incorrect responses		
GPT 3.5	0.096 (0.0584)	0.062 (0.0624)	0.304	<.001
GPT 4	0.194 (0.0211)	0.120 (0.120)	0.682	<.001
GPT 4o	0.193 (0.0226)	0.136 (0.136)	0.590	<.001

^a^Point-biserial correlation between response consistency and response correctness for the models.

^b^Significance set at α=.01 after Bonferroni correction.

In contrast to these findings, there were no associations between the self-reported confidence and response correctness of GPT-4 and GPT-4o (*P*=.98 and *P*=.32, respectively) and only a weak positive correlation between the self-reported confidence and response correctness of GPT-3.5 (*r*=0.211; *P*=.009; Table 4). GPT-4 appeared to generate lower self-evaluation measures and a wider range of scores compared to GPT-3.5 and GPT-4o. GPT-4 assigned self-confidence scores of <20 to the majority of questions it answered both correctly (82/126, 65.1%) and incorrectly (15/24, 63%). GPT-3.5 assigned self-confidence scores of >70 to 98% (62/63) of incorrectly answered questions and 98% (85/87) of correctly answered questions. GPT-4o rated all incorrect and correct questions with self-confidence scores of >70.

**Table 4:** Model self-reported confidence as a predictor of correctness.

Model	Self-confidence, mean (SD)		Correlation coefficient^a^		*P* value^b^
	Correct responses	Incorrect responses			
GPT 3.5	72.8 (4.68)	70.9 (7.69)	0.211	.009	
GPT 4	30.8 (42.3)	24.1 (39.0)	–0.00356	.98	
GPT 4o	82.0 (6.76)	81.8 (6.92)	–0.082	.32	

^a^Point-biserial correlation between self-reported confidence and response correctness for the models.

^b^Significance set at α=.01 after Bonferroni correction.

## Discussion

### Principal Findings

GPT-4 and GPT-4o displayed superior accuracy and greater response consistency compared to GPT-3.5 on standardized psychiatry MCQs. MCQ response consistency displayed a moderate to strong positive correlation with MCQ accuracy across all models. With the exception of GPT-3.5, model self-reported confidence showed no correlation with accuracy.

Advancing the application of generative AI within mental health settings requires that these tools be both accurate and reliable. As a step toward this clinically relevant goal, this study is the first to systematically evaluate the relative performance of 3 common LLMs in demonstrating psychiatric parametric knowledge across a range of indices.

For our first aim, which was to evaluate the relative accuracy of each of the tested LLMs, we determined that the most recently developed models (GPT-4 and 4o) were robust in correctly answering questions reflecting psychiatric knowledge and significantly outperformed their predecessor (GPT-3.5). As a reference point for interpretation, the GPT-4 and 4o models performed at or above the average fourth-year psychiatry resident from our institution on the annual Psychiatry Resident In-Training Examination, whereas GPT-3.5 scored more than 10 percentage points below that benchmark. This finding confirms prior work that has demonstrated the superior performance of GPT-4 models on standardized testing across multiple medical and nonmedical domains [27]. The raw performance of GPT-4 has been somewhat mixed across studies [19]. The psychiatry MCQ accuracy displayed by GPT-4 and GPT-4o (84% and 87%, respectively) is comparable to previously reported GPT-4 accuracy rates in other specialties, such as ophthalmology (82%) and neurosurgery (83%) [37,38]. These findings show promise for the application of LLMs in clinical mental health contexts. Further research is needed to evaluate their performance in more clinically relevant psychiatric scenarios (eg, less structured questions and multiple diagnoses) and investigate potential biases in how LLMs generate and apply knowledge.

While the technical reasons for the performance gains of GPT-4 over GPT-3.5 remain opaque, we may speculate that it is related to GPT-4 being a potentially larger model trained on larger and more representative datasets. Few studies have examined the performance of GPT-4o in medical domains, and the technical reasons it performs on par with GPT-4 are similarly opaque. We can speculate that OpenAI achieved cost and speed gains in GPT-4o (eg, smaller model and training on a more curated dataset) and preserved the psychiatric parametric knowledge encoded within the model.

For our second aim, which was centered on evaluating the reliability of the tested LLMs in psychiatric domains, we found that later models (GPT-4 and GPT-4o) provided more consistent answers to the same MCQs compared to GPT-3.5. Although subject to the same caveats outlined above, these findings suggest that GPT-4 and GPT-4o may be better suited for supervised or autonomous applications in mental health. Their consistency could offer stronger assurances regarding system safety, thereby supporting their potential use in clinical decision support, patient and provider education, and therapeutic chatbot applications.

The greater accuracy and reliability of later models (GPT-4 and GPT-4o) compared to their predecessor (GPT-3.5) contribute to existing literature suggesting LLMs that perform better on general language tasks tend to display similarly superior performance in domain-specific tasks, such as psychiatry. This property is promising as we would expect a future state-of-the-art model (eg, o1 or a future “GPT-5”) to outperform top models available today in terms of mental health-related text interpretation and generation. The finding of no discernable differences in performance between GPT-4 and its more cost- and speed-efficient successor, GPT-4o, in this study suggests that novel optimization techniques for maintaining the general functionality of LLMs while improving their speed and reducing their computational demand and cost may also extend to mental health applications. In other words, methods that make these models more efficient may do so while preserving psychiatric knowledge. If this finding translates to similarly optimized models in the future, it will enable more cost-effective and effective LLM-based mental health services.

Finally, our third aim investigated features that could predict the accuracy of LLM responses to psychiatric questions. Such predictors could help quantify the “confidence” that users should have regarding the accuracy of an LLM’s responses. This would enable technology companies, health care providers, and patients to better determine when close, critical verification of model outputs is particularly important. As a result, they could reduce the risk of a halo effect or “illusions of explanatory depth” [39], which may occur when an LLM initially provides accurate responses, leading to undue trust in subsequent outputs.

Our results suggest that response consistency is a promising predictor of accuracy. When an LLM consistently selected the same MCQ answer across trials, it was significantly more likely to answer the question correctly on its first attempt. On the other hand, when an LLM frequently changed its answers between trials, it was more likely to answer incorrectly on its first attempt. These findings suggest that response consistency can be a valuable metric for estimating confidence scores in LLM responses. To capitalize on this potential, developers of future LLM tools should explore generating multiple responses for each query to enable the measurement of response consistency and inform confidence scoring. Integrating such confidence scores into both supervised and autonomous applications of LLMs will be essential for ensuring feasible, accurate, and safe integration into clinical decision-making not only in psychiatry but also across broader medical fields. In contrast, we found that LLM self-reported confidence did not reliably correlate with accuracy. This is in line with prior research showing that LLMs vary in their ability to accurately predict their own performance. While emerging methods may address these limitations (eg, generating more accurate self-appraisal through reinforcement learning) [40], it is important to emphasize to both the general public and clinicians that the self-confidence responses of LLMs should not be taken as indicators of their actual competence.

Our findings generally indicate that accurate and reliable psychiatric parametric knowledge is encoded in more recent generations of LLMs (GPT-4 and GPT-4o) and support previous work suggesting that the medical parametric knowledge of LLMs has improved over time [19]. These findings provide foundational evidence that state-of-the-art LLMs may be sufficiently advanced that they now demonstrate promise for potential application in clinical settings. Nonetheless, we acknowledge that the structured MCQ format, which has been used to evaluate LLMs across a range of different medical content areas and subfields, is far more structured and unambiguous than practical, clinical scenarios. In addition, there are serious risks of LLMs in psychiatric contexts (eg, LLMs encouraging suicide [41]) and ethical considerations (eg, patients forming bonds with LLMs [42]) that are unexplored in this paper. We believe that there is value to both structured, unambiguous benchmarking tasks (eg, answering MCQs) and more practically applicable but equivocal tasks (eg, case formulation from the “History of Present Illness” section of a clinical note). We view this study as the first incremental step toward elucidating psychiatric knowledge in existing LLMs and recognize the need for subsequent work in several key areas: (1) exploring how to adapt LLMs to perform clinically relevant mental health tasks and applications, (2) investigating the potential serious risks of harm these models could have in mental health contexts, (3) exploring the ethical considerations of introducing AI-based tools into mental health practice, and (4) developing methods to measure and ensure the safety of these models when they operate semiautonomously or autonomously.

### Strengths and Limitations

The strengths of this study include its novel focus on evaluating psychiatric knowledge of LLMs and comparative analysis of 3 different GPT models, which may improve generalizability to other families of models. Furthermore, to our knowledge, this is the first study to examine the reliability of LLMs in a psychiatry context, providing initial evidence for features that could be used to develop a “confidence” measure of LLM responses.

Limitations include a small and relatively uniform dataset (ie, only 150 MCQs from a single source). It will be important for future research efforts to include more representative data, including from multiple sources. Further, it is possible that outdated or biased questions in the underlying MCQ dataset, which was published in 2020 prior to the introduction of the Diagnostic and Statistical Manual for Mental Disorders, 5th edition, Text Revision (DSM-5-TR), skewed the results and deflated accuracy measures. In addition, because the MCQs were publicly available online before the GPT models (GPT-3.5, 4, and 4o) were trained, it is possible that these questions were included in the models’ training data and that the models may have “memorized” the answers during training. This important limitation is shared by several other papers examining the performance of GPT on MCQs [37,43]. Both theoretical and empirical work suggests that models with more parameters have a greater capacity for memorization [44,45]. Thus, some of the performance gains of GPT-4 and GPT-4o over GPT-3 may be attributable to memorization. However, other empiric work has suggested that verbatim memorization is more likely for sequences that are repeated throughout training data [46], which may be less true of highly specialized, psychiatry MCQs. Finally, the structured nature of the MCQ task differs considerably from less structured clinical scenarios where enthusiasts envision LLMs could operate. It will be important for future work to evaluate the performance of LLMs in more open-ended and applicable settings.

This study focused specifically on 3 models: GPT-3.5, GPT-4, and GPT-4o. While these models were selected because of their commercial popularity and breadth of prior research, we did not test LLMs from other providers (eg, Anthropic) or more recent GPT-family models. As future models are released, it will be important to benchmark their performance on psychiatry tasks.

Prompt optimization was beyond the scope of this study, but methods like role prompting, few-shot learning, or chain-of-thought reasoning could potentially improve the accuracy or consistency of GPT models in answering psychiatry MCQs. For example, role prompting (ie, explicitly instructing the model “you are a board-certified psychiatrist who answers questions in line with the latest scientific evidence”) might cue the model to draw on more appropriate domain-specific knowledge and clinical reasoning. Chain-of-thought prompting that encourages models to reason through steps (eg, summarize major symptoms and then build a differential diagnosis) may improve model reasoning for more complex, multistep questions. Given that optimal performance is necessary for any real-world application, future work could focus on developing prompting approaches that maximize accuracy within psychiatry. Finally, self-confidence was assessed preresponse (as opposed to asking the model to rate its generated answer alongside the MCQ). It is possible that the latter approach or other unexplored approaches may improve self-confidence measures.

In our methodology, the model was provided a list of its previous answers to the MCQ when attempting to answer the question again. This design was primarily employed to introduce variability in input and facilitate the measure of inconsistency within a limited number of trials. It is possible that this methodology increased variance in our study (ie, by adding variability to the input prompt, there is more variability in the output). However, this technique may also have allowed the model to engage in a process akin to self-reflection, which may improve accuracy [47]. In addition, it is possible that this approach mirrors the conversation context of multiturn conversations that average patients and health care professionals may use when interacting with LLMs (eg, asking a question in several different ways within a single chat session such that the model has access to its previous responses). In summary, we cannot rule out that this design may have affected the results. Future work could explicitly compare the accuracy and consistency of MCQ answers across three types of contexts: (1) fully independent contexts with no information sharing between queries, (2) contexts like ours with select information sharing between queries, and (3) conversational contexts with full information sharing between queries. However, given that this approach was consistent across models and trials, we believe our comparative conclusions remain valid.

Significant hurdles remain in realizing AI-based mental health tools, including ensuring that LLMs produce accurate information in real-world scenarios, building LLM tools with robust safety, and developing methods to measure and optimize equity across patient groups in LLM performance. Future work should focus on assessing LLM performance in more open-ended psychiatric tasks such as responding to patient or provider questions, developing measures of confidence in LLM responses, and examining bias encoded in LLM representations of psychiatric knowledge. Our findings support the idea that response consistency may serve as an indicator of response accuracy, which may further serve as an important safeguard for integrating LLMs into clinical workflows. In addition, further work is needed to determine the parametric knowledge of LLMs in subspecialties of psychiatry, including child and adolescent psychiatry, geriatric psychiatry, and consult-liaison psychiatry.

### Conclusions

This work establishes that current LLMs encode accurate and reliable psychiatric knowledge and suggests that response consistency may be a useful metric for methods aimed to assess confidence in LLM responses. Moreover, our findings suggest that industry advancements in model quality and cost optimization are applicable to psychiatric use cases, indicating that future models may perform even better in mental health applications than those tested here. These findings suggest that there is significant promise for LLM tools in mental health settings, such as assistance with clinical decision-making, patient education, and chatbot-based treatment augmentation. Such innovations have the potential to dramatically expand access to mental health services and alleviate the severe shortage of psychiatric providers.

## Appendix

Multimedia Appendix 1 Example prompts and GPT responses for a single multiple-choice question.

Multimedia Appendix 2 Response consistency histograms.

## Abbreviations

AI: artificial intelligence
LLM: large language model
MCQ: multiple-choice question

## Data Availability

The data used in this study are publicly available and were accessed in compliance with the fair use clause for research purposes. They were processed through an enterprise OpenAI application programming interface that neither stores data nor uses it for the training of future models. All datasets supporting the findings are obtainable from publicly accessible sources. Detailed information about the data sources and how to access them can be obtained from the corresponding author upon request. The source code used to produce the results can be obtained by contacting the corresponding author.
