# Supplementary material for: Assessing the Accuracy and Reliability of Large Language Models in Psychiatry Using Standardized Multiple-Choice Questions: Cross-Sectional Study
Source: J Med Internet Res. 2025 May 20;27:e69910. doi: 10.2196/69910 (PMC12134693; doi:10.2196/69910)
Supplement: Multimedia Appendix 1 [file jmir_v27i1e69910_app1.docx]

**Multimedia Appendix 1:** Example prompts and GPT responses for a single multiple-choice question.

**Self-Confidence Prompt:** Considering the scope of your training and the data you've encountered, could you assign a numerical value, from 0 to 100, representing the likelihood that your response to the upcoming question will be both accurate and relevant? Please provide the numerical value FIRST and then offer any explanation in a separate paragraph. For example, your response might be: "50%. This estimation considers the factual and objective nature of the question, which aligns well with the type of data I was trained on."

Please provide a likelihood estimate even if you feel you are incapable of providing one.

Which one of the following is not an inhibitor of CYP450 1A2?

E: Grapefruit juice

B: Tobacco

C: Cimetidine

D: Fluvoxamine

A: Amiodarone

**GPT-4:** 0

**Initial MCQ Prompt:** You will receive a multiple-choice question along with potential answers labeled A through E. Please select the best response and provide only the corresponding letter. If uncertain, choose the answer most likely to be correct based on scientific evidence. Each question will be presented to you multiple times, regardless of the accuracy of your previous answers. When available, your previous answers to each question will also be provided.

Which one of the following is not an inhibitor of CYP450 1A2?

E: Grapefruit juice

B: Tobacco

C: Cimetidine

D: Fluvoxamine

A: Amiodarone

**GPT-4:** E

**Subsequent MCQ Prompt:** You will receive a multiple-choice question along with potential answers labeled A through E. Please select the best response and provide only the corresponding letter. If uncertain, choose the answer most likely to be correct based on scientific evidence. Each question will be presented to you multiple times, regardless of the accuracy of your previous answers. When available, your previous answers to each question will also be provided.

Which one of the following is not an inhibitor of CYP450 1A2?

E: Grapefruit juice

B: Tobacco

C: Cimetidine

D: Fluvoxamine

A: Amiodarone

Here are your previous answers: E, E. Please check your work to ensure accuracy and provide your final answer, noting that the initial responses could be either correct or incorrect.

**GPT-4:** B
