# Supplementary material for: Assessing the Accuracy and Reliability of Large Language Models in Psychiatry Using Standardized Multiple-Choice Questions: Cross-Sectional Study
Source: J Med Internet Res. 2025 May 20;27:e69910. doi: 10.2196/69910 (PMC12134693; doi:10.2196/69910)

**Multimedia Appendix 2.** Response consistency histograms. Histogram of response consistency by model and response correctness. Higher response consistency indicates that the model displayed more consistent selection of answer choices, while lower response consistency indicates that the model frequently changed its answer to the multiple-choice question (i.e., it “flip-flopped” between answers). A consistency score of 0.2 indicates that the model chose the same answer across all 10 trials. The y-axis ranges have been standardized to [0, 1] across all graphs to enable comparison.


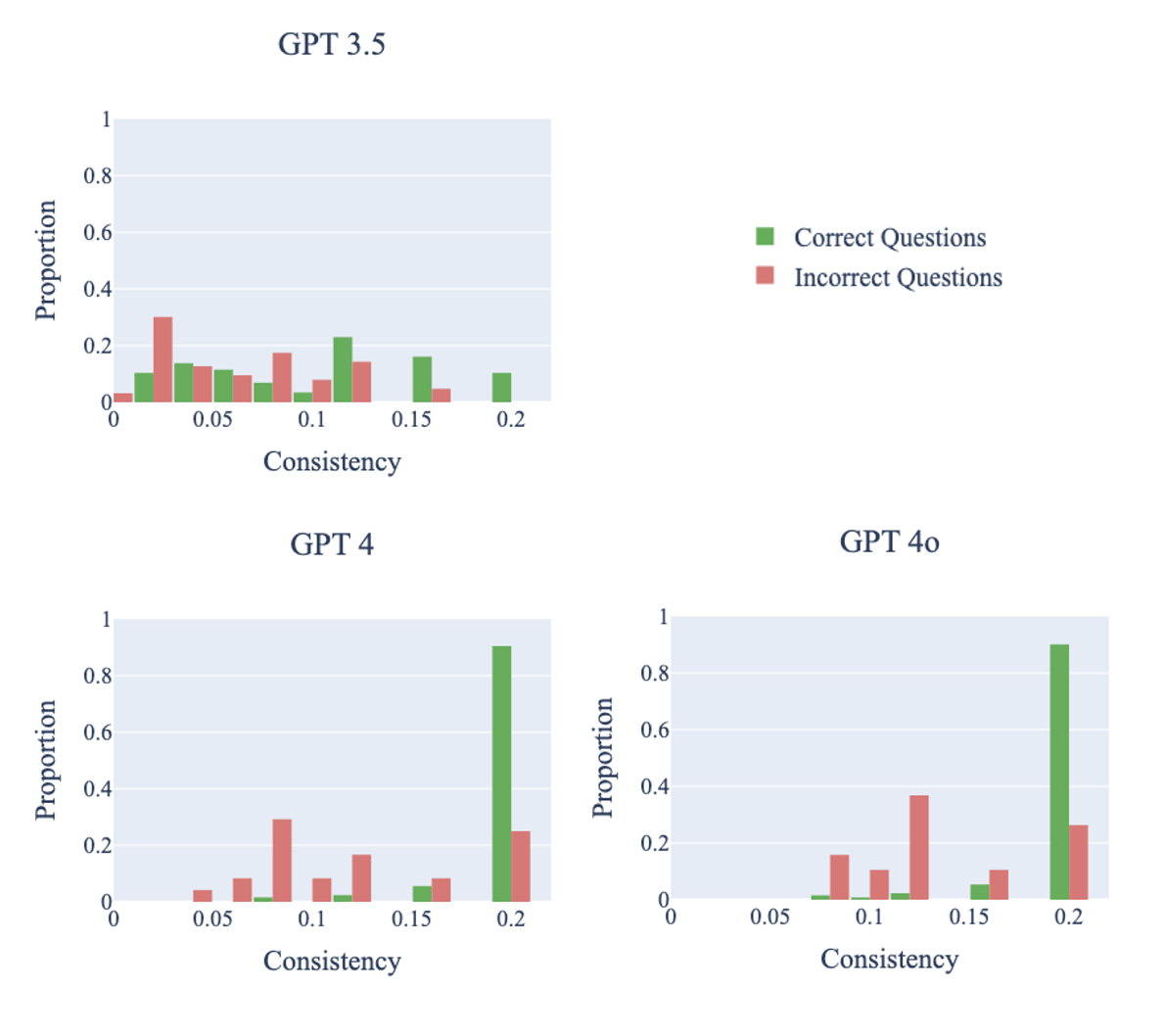

Supplement: Multimedia Appendix 2 [file jmir_v27i1e69910_app2.docx]
